# SnackTrack—An App-Based Tool to Assess the Influence of Digital and Physical Environments on Snack Choice

**DOI:** 10.3390/nu15020349

**Published:** 2023-01-10

**Authors:** Eva Valenčič, Emma Beckett, Clare E. Collins, Barbara Koroušić Seljak, Tamara Bucher

**Affiliations:** 1 School of Health Sciences, College of Health, Medicine and Wellbeing, University of Newcastle, Callaghan, Newcastle, NSW 2308, Australia; 2 Food and Nutrition Program, Hunter Medical Research Institute, New Lambton Heights, Newcastle, NSW 2305, Australia; 3 Computer Systems Department, Jožef Stefan Institute, 1000 Ljubljana, Slovenia; 4 Jožef Stefan International Postgraduate School, 1000 Ljubljana, Slovenia; 5 School of Environmental and Life Sciences, College of Engineering, Science and Environment, University of Newcastle, Callaghan, Newcastle, NSW 2308, Australia

**Keywords:** digital nudging, user interface, food environment, choice behaviour, snacks, snack choice, food choice

## Abstract

As food choices are usually processed subconsciously, both situational and food environment cues influence choice. This study developed and tested a mobile app to investigate the association between physical and digital environments on snack choices. SnackTrack was designed and used to collect data on the snack choices of 188 users in real-life settings during an 8-week feasibility trial. The app asks users to take a photo of the food they are planning to consume and to provide additional information regarding the physical environment and context in which this food was eaten. The app also displayed various user interface designs (i.e., different background images) to investigate the potential effects of images on snack choice. Preliminary results suggest that the time of snack obtainment did not have a significant effect on the healthfulness of the snacks chosen. Conversely, it was found that unhealthy background images appeared to encourage healthier snack choices. In conclusion, despite consumers having the knowledge to make healthy choices, environmental cues can alter food choices. SnackTrack, a novel tool to investigate the influence of physical and digital environments on consumers’ food choices, provides possibilities for exploring what encourages (un)healthy eating behaviours.

## 1. Introduction

Consumer behaviour, including food and nutrition choices, is influenced by factors at the individual and environmental levels. However, the DONE interactive framework [1] demonstrates that environmental factors have the greatest influence on food choices, and importantly, they can be modified. Therefore, strategies to improve dietary habits that are linked with food choices need to focus not only on individual behaviours but also on the food environment and the conditions in which people live and make food choices [2].

Some environmental factors shown to influence food choice and amounts consumed include availability, the effort required for consumption [3], the variety [4] and portion sizes [5] of food presented. Hence, strategically restructuring physical and digital food choice environments appears to be a promising avenue to improve dietary habits and promote population health [6,7]. Modifying the environment by making healthier choices easier and more accessible may have a positive influence on consumers’ food choices. This approach has been described as nudging, which is defined as “any aspect of the choice architecture that alters people’s behaviour in a predictable way without forbidding any options or significantly changing their economic incentives.” [6].

An individual’s health behaviour is determined by conscious and systematic intentions, as well as by automatic, nonintentional and subconscious processes [8]. While habits, including eating habits, can be difficult to change at the individual level, they are affected by environmental cues, such as the visibility of food, which are frequently processed unconsciously. Thus, environmental cues, which are processed outside of conscious awareness, could help in targeting automatic, effortless and spontaneous processes and therefore nudge healthier food choices [9,10,11,12]. One such potential cue is imagery, which carries messages which viewers decode and react to [13]. Food imagery, particularly, may increase food-related thoughts, cravings, hunger, guilt, appetitive appeal, and motivation to eat [11]. It has been shown that images influence consumers’ trust and ease of use of websites [14]. However, to date, few studies have investigated if and how imagery influences food choices [15,16,17,18,19].

In recent years, the consumers’ everyday food choice environments shifted, with food choices now regularly conducted in digital settings, such as online grocery stores, pre-ordering systems, food delivery services, etc. In contrast to traditional food choice environments, the perception of visual cues in a digital setting is entirely mediated through a user interface (UI). As such, UI design may play an important role in consumer perception and food selection. This raises the importance of research in digital nudging, which is defined as “the use of user-interface design elements to guide people’s behaviour in digital choice environments” [20]. A recent review showed that digital nudging is a rapidly growing field, increasingly investigated in food and health contexts [21]. Therefore, the scope of a feasibility study presented in this paper pertains specifically to the imagery used in digital UI and its influence on users.

To date, little is known about how people make food choices within digital environments and which key elements of the UI influence food perception, selection and purchase [7]. Although information and communication technologies are commonly used in the healthy-eating context, research on online tools for guiding consumers’ decision-making processes in the context of healthy food choices is lacking [22]. Additionally, the lack of empirical evidence limits our understanding of how imagery could be used to nudge healthy food choices and if it may even lead to unhealthy food choices.

The frequency of snacking [23] and the contribution of snacks to total energy intake has increased. Research shows that more than 30% of energy comes from snack occasions among Australian children [24] and that snacking contributes to almost one-quarter of total energy intake among Canadian adults [25]. Since the impact of snacking on the overall diet quality depends on their nutritional composition [26], in this study, we specifically focused on snack choices. Snacking is typically defined as the consumption of food or drink between main meals [27]. However, the interpretation of the terms ‘snacks’ and ‘snacking’ may vary by country [28].

In order to understand the contribution of environmental factors and determine the triggers for snack choices, we were interested in tools allowing participants to repeatedly report their experiences in real-time and real-world settings. This kind of report is called Ecological Momentary Assessment [29]. As we could not find a publicly available tool to efficiently investigate environmental and nudging impacts on food choices, the aim of this study was to develop and test a mobile app [30] to assess the contribution of physical and digital environments (i.e., digital nudging). The app allows users to track their snacks while exposing them to different UI designs (implemented as mobile app backgrounds).

## 2. Materials and Methods

### 2.1. Study Design

The feasibility study aimed to design, test and assess a mobile application, and to investigate associations between the digital and the physical environment with snack choices. A mobile app called SnackTrack was designed and developed by the University of Newcastle, Australia, in collaboration with the Computer Systems Department, Jožef Stefan Institute (JSI). SnackTrack was used to collect data on consumers’ snack choices in real-life settings, and the study involved an 8-week feasibility trial to investigate which environmental factors are associated with snack choices. The app enables users to take a photo of the food they are planning to consume via the phone’s camera and allows individuals to provide additional information regarding the physical environment and context in which the food was consumed by selecting pre-set options (e.g., when and how was it obtained, where was eaten, etc.). The whole process of submitting photo(s) and additional information was developed to be as intuitive as possible and not too demanding for app users. For example, participants did not have to enter text or write the answers; they only had to select the most appropriate ones.

The development of SnackTrack focussed on designing a mobile app with a variable UI (in this case, background images) to investigate whether UIs can be used to nudge consumers/app users. In the present study, the goal was to create an app which could be used to investigate whether different mobile backgrounds could nudge consumers towards selecting healthier snack options. Four different backgrounds were implemented and randomised to participants in the intervention conditions. The backgrounds were real-life photos related to either healthy (fruits, vegetables) or unhealthy (sweets, salty snacks) foods (See Figure S1 in Supplementary Materials). These photos were selected because if we wanted to ensure primes will influence consumers and encourage healthy behaviours, environmental cues need to be congruent with health-relevant concepts and products and need to complement the other components in the triggered environment [11]. As nudging should be subconscious, there was no mention of or emphasis on the presence of the backgrounds. Participants allocated to the Control condition used the app without an image background (grey plain-coloured background).

Ethical approval for this study was obtained from the University of Newcastle Human Research Ethics Committee (Approval number H-2020-0267).

### 2.2. Participants and Procedure

Participants were recruited in the period from February to April 2022 via social media and printed posters (displayed at JSI and on campus at the University of Newcastle). Individuals interested in participating in the study scanned a QR code on the recruitment material to download the mobile app SnackTrack from either the Apple App store or Google Play. SnackTrack was only available in the app stores in Australia (English version) and Slovenia (Slovenian version). After downloading the app, participants received a study information statement, and consent was obtained online prior to the data collection. Each participant received a unique code to protect their privacy; thus, no information on identity was recorded during the research study. Before being able to use the app, participants provided information about their sex and year of birth. Only participants aged 18 years or older were eligible to participate.

Participants were randomised into 5 conditions (4 intervention conditions; mobile background containing photos of either (i) fruits, (ii) vegetables, (iii) salty snacks or (iv) sweets, and a Control condition (v); grey-coloured background). Randomisation was performed directly in the app, using an algorithmic approach for randomisation to avoid any subjective decision. Each background was assigned once per cycle; therefore, each 5th participant downloading the app was allocated to the Control condition. Participants were asked to take photos of the snacks they consumed or were planning to consume throughout the day. Participants were instructed to capture the photo of the foods as close as possible and to make sure that the food was clearly visible. Photos could be taken of the snacking occasion (e.g., multiple food items on one photo,) or each food item could be captured separately. Photos submitted at the same time were treated as one snacking occasion. Together with photos of snacks, additional data about snacking choices, such as how and when the snack was obtained and where and in the company of how many people the snack was consumed, were collected. A screenshot of the additional data screen with the vegetable background is shown in Figure S2 in Supplementary Material. Participants were asked to upload at least 15 photos over 15 days between February and April 2022. Therefore, they were asked to use the app at least once a day for at least 15 days (no need to be consecutive). Participants who submitted at least 15 photos of actual foods were eligible to be included in a prize draw for either a $50AUD gift card (Australian participants) or a €10 gift card (Slovenian participants).

### 2.3. Sample Size

We aimed to recruit 500 participants (250 from each country, 50 in each condition), which is the number needed to detect medium to large effect sizes. We anticipated recruiting as many participants as possible within the eight weeks of the intervention.

### 2.4. Measures

After data collection was completed, we checked all the photos and removed duplicates. Duplicates could have been uploaded if the internet connection was lost while submitting the photo (this resulted in uploads of multiple of the same photos on the server). In some cases, the photos were not uploaded to the server correctly, and we could not open and see the photos. These photos were also removed from the analysis. After the removal of duplicates and ‘broken’ photos, we annotated and described each photo (snack). We annotated the name of the food on the photo (e.g., chocolate bar), the Nutri-Score (NS) [31] of the foods, and the food group the food corresponds to. The food groups used, together with examples based on submitted photos, are shown in Table 1.

The primary outcome variable was the average NS [31,32,33], which is a colour-coded index of overall diet quality ranging from A (healthiest) to E (least healthy) based on the British Food Standard Agency Nutrient Profiling System. For each snack (photo), we applied the standard Nutri-Score algorithm to assign a score from A to E based on nutritional quality. Later, we recorded the scores as numbers from 5 (A: the healthiest) to 1 (E: the least healthy). We calculated an average score for the snacking occasion in the following cases: when the photo contained multiple foods (e.g., mandarins and chocolate bar) as a part of one snacking occasion or if participants submitted more than 1 photo, but it was clear that the photos were part of one snacking occasion (e.g., on 1 photo a hot cross bun, and on the other a handful of nuts; both photos were submitted together).

In addition, two independent accredited dietitians (one from Slovenia and one from Australia) were asked to assess the healthiness of the photos. They assigned a score from 1 (very unhealthy) to 5 (very healthy). The dietitian’s holistic assessment score (DA) was needed because NS could not be applied to all of the snacks, as some photos did not contain the information needed to assign the score. For example, foods prepared and self-packed in advance did not contain the dietary labels needed to calculate the NS (e.g., we could not distinguish the fat content of dairy products).

### 2.5. Data Analysis

Descriptive statistics were used to describe the properties of participants and the additional data about snacking choices (when and where the snacks were obtained, where they were eaten and with whom) using Microsoft Excel and R software [34] (v. 4.2.0, for iOS). Demographic details were summarised with frequencies, means, and standard deviations (SD) as appropriate. The time of the photo submission was considered as the time of the snack consumption. For data analysis, time was corrected to correspond to either CEST or AEST time zone (Daylight saving time change was taken into consideration), depending on the participant’s location.

Data were tested for normality with the Shapiro–Wilk test, and the homogeneity of variances was tested using Levene’s test, using R software; *p*-values < 0.05 were considered statistically significant. Statistical comparison of the differences in NS and DA between conditions was performed using a Kruskal–Wallis test, followed by Dunn’s posthoc test (*p*-value adjustment method; Benjamini and Hochberg). A chi-squared test was used to compare the proportions of food groups in the 5 conditions.

## 3. Results

### 3.1. Participants

A total of 284 adults downloaded the app; of those, 80 were from Australia and 204 from Slovenia. Of the 284, 95 people had not submitted any photo, and one person entered invalid data for their year of birth. Therefore, data from 188 people (52 from Australia and 136 from Slovenia) were included in the final analysis. Out of those, 64 people submitted 15 photos or more, and the average number of days participants were using the app was 5.02. Participants were randomised in Control (*n* = 36), ‘Fruit’ (*n* = 47), ‘Vegetable’ (*n* = 34), ‘Salty snacks’ (*n* = 36), and ‘Sweets’ condition (*n* = 35). Participants in the Control condition submitted 353 photos; in the ‘Fruit’ condition, 441 photos; in the ‘Vegetable’ condition, 297 photos; in the ‘Salty snacks’ condition, 354 photos; and in the ‘Sweets’ condition, they submitted 318 photos.

The mean age of the participants was 35.5 years (±12.8); of those, 72.3% identified themselves as female (*n* = 135), 25% as male (*n* = 47), 2.1% as other (*n* = 4), and 1.1% declined to answer (*n* = 2).

### 3.2. Feasibility Outcomes

**Figure 1:**
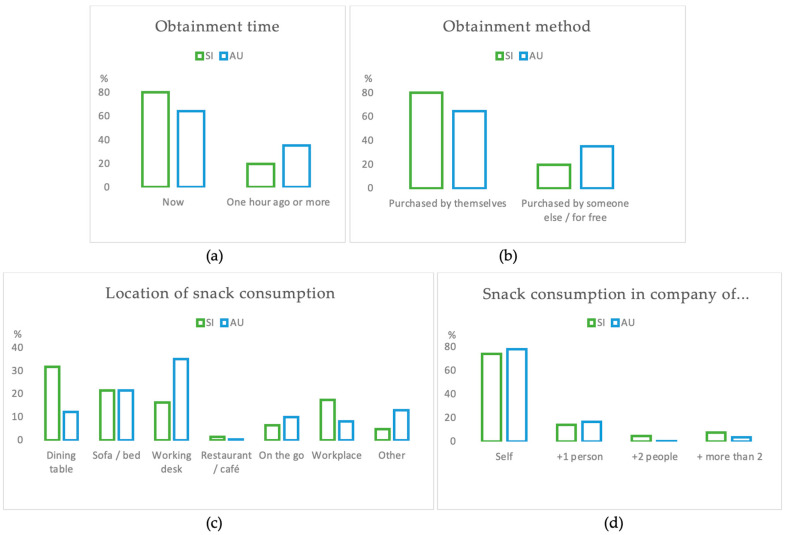
Results on the context of snack consumption: (**a**) Time of snack obtainment; (**b**) Method of snack obtainment; (**c**) Location of snack consumption; (**d**) How many people participants were with while eating the snacks.

During the intervention, participants submitted a total of 1763 (1297 from Slovenia and 466 from Australia) photos of snacks. Of those, 76.1% (*n* = 1343) were obtained at the moment of consumption (80.3% (*n* = 1042) from Slovenia and 64.6% (*n* = 301) from Australia) and 23.8% (*n* = 420) were obtained more than one-hour prior consumption (19.7% (*n* = 255) from Slovenia and 35.4% (*n* = 165) from Australia). Most snacks were purchased by the participants themselves (66.3%; *n* = 1169), whereas 33.7% (*n* = 594) of snacks were purchased by someone else, or participants got them for free. The majority of snacks were consumed when participants were by themselves (74.9%; *n* = 1322), followed by when they were accompanied by one additional person (14.5%; *n* = 257), more than two people (6.5%; *n* = 114), or by exactly two people (4.0%; *n* = 70). Overall, the most snacks were consumed at the dining table (26.6%; *n* = 469), followed by on the sofa (21.6%; *n* = 382), at the working desk (21.3%; *n* = 375), in the workplace (15.0%; *n* = 265), on the go (7.3%; *n* = 129), in restaurants/cafés (1.2%; *n* = 22), and “other” was selected for 6.9% of the snacks. The majority of snacks consumed by Australian participants were eaten at the working desk (35%; *n* = 163), while Slovenian participants consumed the most snacks at the dining table (31.8%; *n* = 412). See Figure 1 for more details.

When the time of the day of the snacking occasion was considered, it was found that 37.6% (*n* = 663) of reported snacks were consumed in the morning (from 5 a.m. to 12:59 p.m.), 36.9% (*n* = 652) in the afternoon (from 1 p.m. to 5:59 p.m.), and 25.4% (*n* = 448) in the evening/at night (from 6 p.m. to 4:59 a.m.). Slovenian participants submitted the most pictures of snacks between 5–6 p.m., while Australian participants between 4–5 p.m. We found that photos of snacks submitted by men are more likely to be consumed in the evening (24.9%) than those of women (18.1%).

The mean NS of all submitted snacks was 2.7 ± 1.6 out of five, and the mean DA was 2.5 ± 1.6 out of five. The nutritional quality of snacks among men was slightly poorer (NS: 2.5 ± 1.7 and DA: 2.4 ± 1.6) compared to women (NS: 2.7 ± 1.6 and DA: 2.5 ± 1.6) but not statistically different (NS: *p* = 0.66; DA: *p* = 0.32). Additionally, the difference in NS and DA between Australians (NS: 3.1 ± 1.6; DA: 2.6 ± 1.6) and Slovenians (NS: 2.5 ± 1.6; DA: 2.4 ± 1.5) was significant (NS: *t*(763) = 6.0612, *p* < 0.001; DA: *t*(785) = 2.6708, *p* = 0.008), meaning that Australians consumed healthier snacks. In addition, the results showed that the difference in NS and DA between snacks obtained at the moment of consumption (NS: 2.7 ± 1.6 and DA: 2.5 ± 1.5) and the ones obtained more than 1 hour before consumption (NS: 2.7 ± 1.7 and DA: 2.5 ± 1.6) was not significant (NS: *t*(697) = 0.45216, *p* = 0.65; DA: *t*(673) = −0.10023, *p* = 0.92), which means that the time of snack obtainment did not affect healthfulness of snacks. Moreover, snacks eaten when participants were alone were significantly healthier that when participants were in company with others (NS: *t*(759) = −7.3674, *p* < 0.001; DA: *t*(885) = −7.9996, *p* < 0.001).

Regarding the time of snacking, a Kruskal–Wallis *H*-test showed that there was a statistically significant difference in NS and DA between the times of day the snacks were consumed (NS: *χ^2^*_(2)_ = 23.85, *p* < 0.001; DA: *χ^2^*_(2)_ = 51.29, *p* < 0.001) with a mean NS/DA score of 2.9/2.7 for morning snacks, 2.7/2.5 for afternoon snacks and 2.4/2.1 for evening snacks.

As the purpose of the app is to assess whether UI design (background) has an impact on the healthfulness of the snacks consumed, we first checked the mean NS and DA for all five backgrounds. The mean NS and DA scores were the lowest in the Control condition (NS: 2.4 ± 1.5; DA: 2.2 ± 1.5) and the highest in the ‘Sweets’ condition (NS: 2.9 ± 1.7; DA: 2.7 ± 1.6) as seen in Table 2. When analysing differences between the conditions (data were not normally distributed (*p* < 0.001) and did not have equal variance (NS: *p* < 0.001; DA: *p* < 0.005)) results showed that there was a statistically significant difference in NS (*χ^2^*_(4)_ = 21.418, *p* = 0.0003) and DA (*χ^2^*_(4)_ = 18.392, *p* = 0.001) as presented in Table 2. The posthoc test indicated significant differences in NS of snacks between Control and ‘Fruit’ (*p* = 0.04), Control and ‘Salty snack’ (*p* = 0.004), Control and ‘Sweets’ (*p* = 0.0002), and between ‘Vegetable’ and ‘Sweets’ (*p* = 0.04). See Table 2 for more details and for the condition differences between DA scores.

In addition, which food groups were most represented among the conditions was evaluated. Interestingly, among all five conditions, the majority of photos of snacks corresponded to the sweets food group, followed by fruits. See Table 3 for more details. This indicated that the majority of snacks consumed were chocolate, cookies, candy, fruit/nut/granola/protein bars or similar, followed by different types of fresh fruits, fruit-only smoothies or dried fruits. When comparing between countries, the Slovenian participants submitted more sweet snacks (46.7%) than Australian participants (35%). In addition, one-quarter of Slovenians and over 22% of Australians had fruits as a part of their snacking occasions, and less than 3% of Australians and less than 2% of Slovenians consumed vegetables as snacks. Moreover, background images had a significant effect on snack choices (*χ^2^*_(45)_ = 100.95, *p* < 0.001), and from the visual presentation in Figure S3 in Supplementary Materials, it can be seen that in the ‘Fruit’ condition, more photos of ‘Mixed meals and other’ were submitted, and in ‘Sweets’ condition more photos of ‘Beverages’ were submitted. Post-hoc analysis indicated that only the proportion of snacks within the ‘Mixed meals and other’ condition (Table 1 for more details) food group is significant (*p* = 0.02). There was no significant difference between other intervention groups.

We also investigated whether background images were associated with the selection of foods present in this image. For example, whether fruity background images stimulate fruit consumption. We found only in the ‘Sweets’ condition that the percentage of sweets containing photos was the highest (41.2%) compared to other conditions. For this purpose, all photos containing food(s) corresponding to the background foods were considered. This means that if fruit and cookies were consumed as a part of one snacking occasion, the cookies were considered in the ‘Sweets’ condition, even if not solely cookies were in the photo.

## 4. Discussion

Innovative interventions are needed to understand how people make food choices within digital environments and which (if any) UI features influence the selection the most. This study explored the feasibility of an app-based tool to investigate (physical and digital) environmental influences on snack choice.

In the existing literature [35], it is seen that monitoring dietary intake can be very demanding for people; thus, we wanted to develop an easy-to-use and intuitive app. Hence, we intentionally removed the burden of taking pictures of separated foods or ingredients when consuming a composite dish (e.g., all the ingredients used to prepare the sandwich) and providing additional information by typing. Instead, the app allows selecting pre-set options.

Contrary to the studies suggesting that meal planning and food prepared at home is healthier (e.g., [36,37,38]), in our study, we found that the average NS or DA did not significantly change between snacks obtained immediately prior to consumption and the ones obtained more than one hour before consumption. This may be due to the fact that the intervention was performed during the COVID-19 pandemic when people worked remotely or mainly from home. This would also justify that more than 75% of snacks were obtained at the moment of consumption, as participants did not need to prepare the foods in advance. Similarly, it would explain the fact that the majority of snacks were eaten at the dining table (Slovenians) or at the working desk (Australian). In addition, 75% of snacks were consumed when participants were by themselves, which can again be a consequence of the working-from-home requirement. However, in contrast to a study by Chae et al. [39], we found that the food quality of these shacks was higher compared to snacks eaten in the company of others (either one, two, or more people).

We found that among participants from both countries, the majority of snack photos contained some sort of sweets, but photos submitted by Slovenians contained more of these compared to photos from Australians. This might be due to the holiday season in Slovenia, which took place during the data collection when fried sweet pastries are traditionally consumed. In addition, surprisingly, one-quarter of Slovenians and a bit more than 22% of Australians had fruits as a part of their snacking occasions. In contrast, only less than 3% of Australians and less than 2% of Slovenians consumed vegetables as a part of the snacking occasions. A very recent study showed that the majority of Polish adults are aware of the importance of vegetable and fruit intake, the negative effects of the consumption of sugar and salt, and the related dietary risk factors [40]. Nevertheless, many people still do not meet the recommended fruit and vegetable intakes (e.g., [41,42,43]), even though research is consistent that fruits and vegetables have beneficial effects on our overall physical and mental health [44,45,46]. This calls for innovative strategies to promote nutritious snacks and overall food consumption, which can be challenging as people have difficulties assessing snacks’ nutritiousness when snacks contain healthy and less healthy components (e.g., fruit yoghurt containing large amounts of sugar or nuts with high energy and/or salt content) [47]. Despite the widespread knowledge about the impact of diet on health, people still have difficulties consuming and selecting nutritious foods. Hence, promoting nutritious snack consumption of, for example, snack-sized vegetables (such as mini carrots, which can be found in any well-stocked Australian store) and making them more accessible could increase vegetable intake and reduce health-related risks.

In addition, findings here are consistent with results from other studies suggesting that women make healthier food choices than men (e.g., [48]). Moreover, in agreement with previous studies on adults in France and Australia, our findings suggest that snacks consumed in the morning are healthier than the ones consumed later in the day [49,50]. Further research needs to investigate what drives these behaviours to promote healthier snacking behaviours and choices.

The nudging theory suggests that manipulating the consumer’s environment can change behaviour in a predictable way. Hence, it could be expected that people who are exposed to healthy images would be nudged to choose healthier food [51]. However, our results showed that NS and DA were the highest (i.e., more healthful) in the ‘Sweets’ condition. Moreover, the DA score was the lowest in the ‘Fruit’ and ‘Vegetable’ conditions. This suggests that presenting images containing healthy foods can encourage unhealthy food choices and vice versa; images of unhealthy foods (sweets) may nudge consumers towards healthier food choices. While this is inconsistent with the previous results of other studies conducted in real-life settings (not online) (e.g., [15]), there is literature supporting our findings that healthy cues can lead to unhealthy choices (e.g., [52,53]). In the study by Wilcox et al. [52], consumers that saw a healthy food option on a menu were more likely to choose the least healthy option compared to when a healthy option was not included. This effect is called vicarious goal fulfilment, where the mere presence of healthy options fulfils the need to make healthy food choices and provides the individual with a rationale for unhealthy choices. Therefore, it may be possible that seeing unhealthy foods fulfilled participants’ desire to indulge and select unhealthy snack and thus they felt satisfied enough to make a healthy choice. However, the small sample size within each condition may have influenced these results, which should be interpreted with caution. Additional studies are warranted to determine whether these findings can be repeated in a larger sample size, and our findings provide a rationale for investigating this.

Moreover, results here showed that participants in the ‘Sweets’ condition submitted more than 40% of snacks that corresponded to the sweet confectionary food group. These conflicting results need further investigation, and future research should examine whether the positive benefits of nudging strategies used in real-life settings have the same effect in online settings. For example, can repositioning foods in online settings influence food choices the same as it does in field studies [3]? In addition, as UIs can easily be modified in an online environment, it may be beneficial for our understanding of UI impact on consumers to test different types of imagery. In our study, we used food imagery; however, the potential impact of using nature imagery or warning graphic images (as seen on cigarette packages) in an online setting still needs to be addressed. Hence, SnackTrack is an open-source mobile app, and its source code is freely available (See Computer Codes S1–S3 in Supplementary Materials) for use and modification required for any research purposes (CC-BY-NC, i.e., the Creative Commons Attribution-NonCommercial). 

### Limitations

The current study has some limitations that need to be acknowledged. Firstly, the small sample size prevented us from detecting small effects. Participants overall submitted fewer photos per person than anticipated, impacting the ability to assess the overall impacts of the image-based nudging. This feasibility study confirmed that food intake monitoring could be a burden to consumers, as participants were struggling with tracking their snack intakes for 15 days. This might also be due to the fact that 25% of apps are used only once after being downloaded [54]. Furthermore, sending reminders to participants to encourage them to track food intake would be advisable, although this cannot guarantee photo acquisition, as reminders are sent automatically and not necessarily immediately before snacking occasion. In addition, the NS nutrient profiling system, as well as the developed app, did not consider the portion size of foods. Since SnackTrack is not a dietary assessment application, portion size estimation was beyond the scope of the study, which was to assess the impact of the UI on food choices. Future research should incorporate portion size into a nutrient profiling system and/or the app and compare nutrient profile scores relative to nudging strategies.

Another limitation may be the understanding of the definition of snacking and the perception of what is considered a snack. For example, in Slovenia, a lot of companies offer warm meals for employees during working hours. Therefore, people frequently eat a multi-course meal (e.g., soup, potatoes with meat and salad) between breakfast and lunchtime. Even though we did not receive many photos of meals like this, we do have to emphasise that ‘a meal between main meals’ might mean different things for different people, cultures, etc.

Lastly, investigating the ability of imagery to nudge consumers over time is warranted. As environmental cues affect eating habits and are processed subconsciously, consumers might have been nudged the first time using the app. Currently, there is no strong evidence of how much time it takes to influence consumers to change their dietary behaviours; therefore, future research should seek to address this. For example, investigating how long the exposure should be for (food) imagery to target and increase cravings and motivation to eat certain foods (e.g., healthy foods) and if the exposure time is different for different types of foods (e.g., healthy vs unhealthy) or during the day. In addition, how the healthfulness of foods or diet quality changes over time while exposed to nudges (e.g., if more and more unhealthy choices were made in the unhealthy condition) needs further investigation. In order to investigate this, monitoring dietary intake and tracking behavioural changes or nutritional quality of the snacks over a period of time is needed. Hence, finding a solution to motivate participants to track their food intake is warranted. A potential solution could be automatic food image recognition [55,56], which can further contribute to the development of automated dietary assessment. Future research should seek to address this. Lastly, we would like to emphasise that during the recruitment period, COVID-19 lockdown restrictions were in place in both countries.

## 5. Conclusions

This pilot study demonstrates that food (online) environments play an important role, and innovative strategies are needed for behavioural changes. Since promoting healthy foods can impact consumer choices and have the opposite effect than intended, our findings suggest that future research needs to address this, especially in an online context. Further, a clear description of implemented UI elements to investigate and quantify the effect on consumers’ food choices is needed. In addition, while consumers have the knowledge to make a healthy choice, the plethora of environmental stimuli influencing them is evidently altering food choices. Although the current study has a relatively small sample size, it still provides some interesting findings, such as insights into the healthy cues leading to unhealthy choices and the time of food obtainment not having an effect on the healthfulness of selected foods.

## Figures and Tables

**Table 1:** Food groups with examples of foods corresponding to them.

Food Group	Examples of Foods in the Food Group
Fruits	Fresh fruits, fruit-only containing smoothies, dried fruits
Vegetables	Fresh and pickled vegetables
Salty snacks	Chips, tortilla chips, popcorn, salty crackers, salty sticks/pretzels
Sweets	Chocolate bars, cookies, candy, granola/muesli/protein/nut/fruit bar, sweet pastries, cakes, waffles, etc.
Grains	Bread bun, sour pastries, rice cakes, etc.
Dairy and dairy substitutes	Yoghurt (natural or flavoured), quark, puddings, plant-based yoghurt
Meat, fish & egg	Cold cuts, jerky, salmon, eggs
Beverage	Juices, coffee/tea (with milk), carbonated drinks, energy drinks, alcoholic beverages
Nut	Nuts (plain, roasted, salted), nuts with dried fruits
Mixed dishes and other foods	Sandwich, toast with spread, soup, pizza, fries, porridge, breaded vegetables, pancakes with spreads, peanut butter, honey, etc.

**Table 2:** Nutri-Score and Dietitian’s Assessment Score per condition.

			Nutri-Score (NS)					Dietitian’s Assessment Score (DA)				
**Conditions**		*n*	Mean (SD)	*df*	*χ^2^*	*p*	Comparison condition	Mean (SD)	*df*	*χ^2^*	*p*	Comparison condition
				4	21.418	**<0.001**			4	18.392	**0.001**	
**Healthy conditions**	Fruit	441	2.7 (1.6)			0.59	Vegetable	2.4 (1.5)			0.39	Vegetable
						0.34	Salty snacks				0.30	Salty snacks
						0.06	Sweets				0.29	Sweets
	Vegetable	297	2.6 (1.7)			0.17	Salty snacks	2.4 (1.6)			0.10	Salty snacks
						**0.04** *	Sweets				0.10	Sweets
**Unhealthy conditions**	Salty snacks	354	2.8 (1.7)			0.30	Sweets	2.6 (1.6)			0.98	Sweets
	Sweets	318	2.9 (1.7)					2.7 (1.6)				
**Control condition**	Control	353	2.4 (1.5)			**0.04** *	Fruit	2.2 (1.5)			**0.03** *	Fruit
						0.16	Vegetable				0.25	Vegetable
						**0.004** **	Salty snacks				**0.003** **	Salty snacks
						<**0.001** ***	Sweets				**0.002** **	Sweets
	Total	1763	2.7 (1.6)					2.5 (1.6)				

SD, standard deviation; *χ^2^*, results from Kruskal-Wallis test; *df*, degree of freedom; * *p* < 0.05; ** *p* < 0.01; *** *p* < 0.001.

**Table 3:** Food groups of snacks per condition.

		Healthy Conditions		Unhealthy Conditions		Control Condition
All photos *n* (%)		Fruit *n* (%) *n^p^* = 47 (AU = 13; SI = 34)	Vegetable *n* (%) *n^p^* = 34 (AU = 11; SI = 23)	Salty snacks *n* (%) *n^p^* = 36 (AU = 9; SI = 27)	Sweets *n* (%) *n^p^* = 35 (AU = 9; SI = 26)	Control *n* (%) *n^p^* = 36 (AU = 10; SI = 26)
**Fruits**	412	103 (25)	67 (16.3)	92 (22.3)	82 (19.9)	68 (16.5)
**Vegetables**	35	5 (14.3)	8 (22.9)	5 (14.3)	13 (37.1)	4 (11.4)
**Dairy**	169	50 (29.6)	25 (14.8)	44 (26.0)	35 (20.7)	15 (8.9)
**Grains**	41	11 (26.8)	6 (14.6)	13 (31.7)	4 (9.8)	7 (17.1)
**Meat, fish and eggs**	18	4 (22.2)	1 (5.6)	7 (38.9)	0 (0.0)	6 (33.3)
**Nut**	130	26 (20.0)	16 (12.3)	27 (20.8)	38 (29.2)	23 (17.7)
**Salty snacks**	158	33 (20.9)	40 (25.3)	31 (19.6)	15 (9.5)	39 (24.7)
**Sweets**	770	190 (24.7)	129 (16.8)	136 (17.7)	141 (18.3)	174 (22.6)
**Beverages**	94	23 (24.5)	7 (7.4)	15 (16.0)	31 (33.0)	18 (19.1)
**Mixed meals and other**	166	64 (38.6)	21 (12.7)	28 (16.9)	20 (12.0)	33 (19.9)

*n* = number of submitted photos; *n^p^* = number of participants from Australia or Slovenia.

## Data Availability

The data presented in this study are available on request. The data are not publicly available due to privacy and ethical restrictions.

## Supplementary Materials

The following supporting information can be downloaded at: https://www.mdpi.com/article/10.3390/nu15020349/s1. Figure S1: Background images used in four conditions; Figure S2: A screenshot of Take a photo screen and the additional info screen with the vegetable background; Figure S3: Visual presentation of standard residuals; Source code S1: SnackTrack – iOS version; Source code S2: SnackTrack – Android version and Source code S3: SnackTrack – Server.
