# Supplementary material for: SnackTrack—An App-Based Tool to Assess the Influence of Digital and Physical Environments on Snack Choice
Source: Nutrients. 2023 Jan 10;15(2):349. doi: 10.3390/nu15020349 (PMC9862135; doi:10.3390/nu15020349)
Supplement: Supplementary file 1 [file nutrients-15-00349-s001.zip › nutrients-2128349-supplementary.pdf]

Supplementary Materials:

Figure S1. Background images used in four conditions. (A) 'Fruit' condition; (B) 'Vegetable' condition; (C) 'Salty snacks' condition and (D) 'Sweets' condition. Images were obtained from Adobe Stock (A: #87157842; B: #405562967; C: #150900521; D: #246425675).

(A)
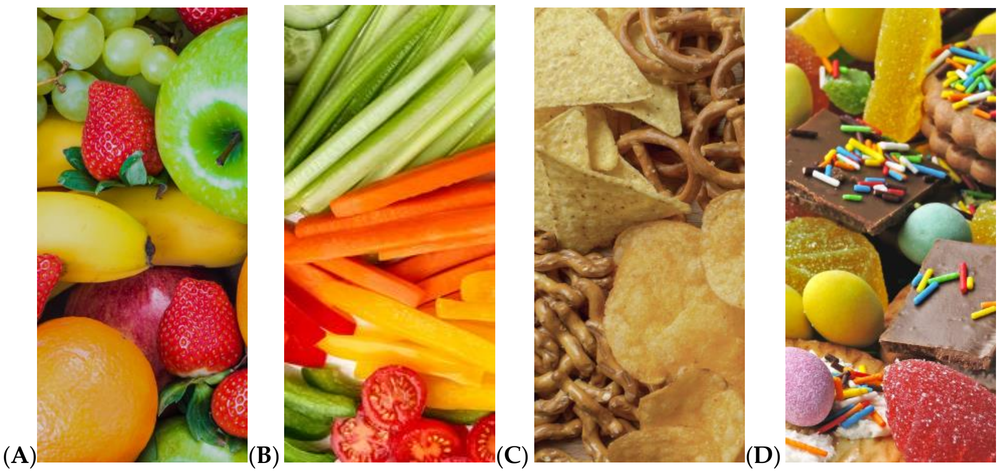

Figure S2. A screenshot of Take a photo screen (left) and the additional info screen (right) with the vegetable background. On the right screenshot "now", "purchased", "self" and "working desk" are selected options.

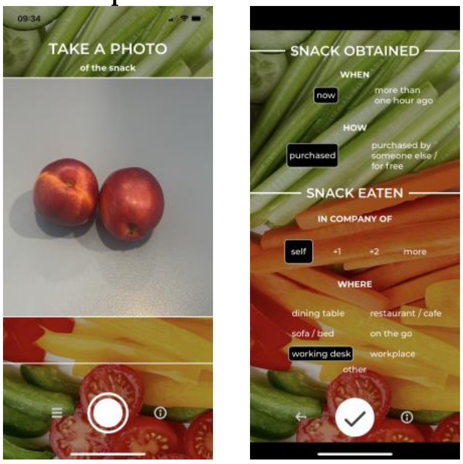

Figure S3. Visual presentation of standard residuals. The size of the circle is proportional to the amount of the cell contribution.

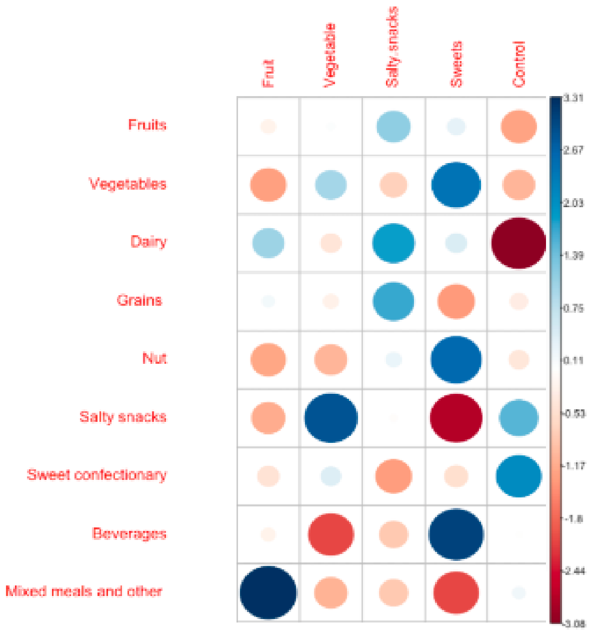

Source Code S1. SnackTrack – iOS version: https://repo.ijs.si/matevzog/snacktrack.

Source Code S2. SnackTrack – Android version: https://repo.ijs.si/andrazsimcic/snack-track-android.

Source Code S3. SnackTrack – Server: https://repo.ijs.si/andrazsimcic/snacktrackserver.
